# Baltimore Alms-House Infirmary

**Published:** 1830-07-01

**Authors:** 


### CLINICAL REVIEW.

### XIII.

#### BALTIMORE ALMS-HOUSE INFIRMARY.

In No. VIII. of the American Journal of the Medical Sciences is a report of cases by Dr. Wright, physician to the above institution. We extract one or two of the more interesting.

#### I. LARYNGITIS.

CASE 1. *Cynanche Laryngea* :—" Samuel Morgan, aged forty-three, tall, spare person, florid countenance, light eyes and hair, admitted January 15th, 1829, suffering under the chronic consequences of what he termed a bad cold, contracted some weeks before. He was very hoarse, almost without distinct voice, short dry cough, not frequent, no pain in any part of the breast, no difficulty either in common breathing, or on making full inspiration. There was no fever in the general, though the patient before coming to the Alms-house, had laboured under an irregular intermittent, which still occasionally recurred. The pulse commonly small, without frequency, and soft, tongue clean, appetite moderate, bowels regular, evacuations of ordinary character, no evidences of gastric derangement. The main features of the case, in short, were made up of the peculiar hoarseness, dry cough, and considerable general debility; tracheitis, now chronic, with some bronchial concern, seemed the primary and still predominant affection, to which all the other symptoms, the general debility, and probably the intermittent also, held a dependent relation."

Under the use of alteratives internally, with counter-irritation over the top of the chest, the patient improved so far as to be able to leave the house in the second week in February.

"On the twenty-fourth of the same month, twelve days after being reported for work, I found Morgan again in the hospital. He was now acutely ill; his face flushed, of a purplish hue, countenance betraying great distress, breathing slow and difficult, often causing a struggle for breath, in which he would raise himself on the bed, throw his head back as far as possible, and bring into forcible action every muscle concerned in opening the chest for admission of air. Just before and after one of those severe struggles for inspiration, he made violent efforts to throw off masses of ropy mucus which seemed to fill the posterior fauces, and choke the passage of the larynx. The entrance and expulsion, particularly the former, of air through the larynx, was attended by a loud sound, of that peculiar rough, ringing character, sometimes called metallic, so eminently characteristic of extreme spasmodic or inflammatory narrowing of the apertures of the glottis and larynx. The act of swallowing was somewhat hindered, and painful, though far less embarrassed than the respiration. The external aspect of the throat, not less than other symptoms, shewed the character, and in some degree the extent, of the affection which had brought the unfortunate patient into so much distress and danger. The body of the larynx in front, with the corresponding portion of œsophagus behind, were swelled out into a globular form of much more volume than the natural state of the parts, the surface of the tumour, and the whole front of the neck, having the same dark red hue as the face, and the enlargement possessing great hardness and sensibility to the touch. The pulse was small, quick, and weak, having neither tension nor firmness. The report furnished by the resident students was, that the man had been brought into the ward the night preceding, with the symptoms just described, but of less intensity; that he was then chilly and depressed, and seemed incompetent to bear active treatment by direct depletion, but had been freely vomited, and had taken a mercurial cathartic."

The smallest-sized cupping-glasses were applied in quick succession to the seat of tumefaction, and a draught of infusion of seneca, tartrite of antimony, in small portions, oxymel of squills, and compound tincture of camphor exhibited at short intervals. The patient also inhaled the vapour of hot water in which chamomile and serpentaria had been infused, with great relief. When the cupping-glasses were removed the scarifications bled freely, and on the subsequent application of a blister the local hæmorrhage was rather considerable. The consequence of this free local depletion was complete relief to the swallowing and breathing, and after a few days cordial nourishment and light tonics were administered with the effect of producing a permanent cure.

CASE 2. *Chronic Laryngitis.*—Margaret M'Carthy, æt. 42, of robust constitution, admitted May 10th, 1827.

"Respiration sonorous, and somewhat laboured, but not painful; voice a hoarse whisper; no fever; cough occurring in paroxysms somewhat severe, without expectoration; pale countenance; not much emaciation; no particular debility, nor other signs of general bad health. In February preceding, she had been attacked by symptoms of croup, as she reported, and was dangerously ill for many days. She recovered slowly, with the alteration of voice, embarrassed respiration, and cough, above described, which symptoms continued ever since, and had undergone occasional exasperation, by cold, fatigue, and other disturbing causes.

"The patient had been but a short time in the infirmary, before an acute attack of her disorder supervened. She had been out of the ward for a few hours in the day, and at night complained of feeling chilly and unwell; in the course of that night her breathing became difficult and painful, with a feeling of great stricture across the top of the thorax; inspiration was impeded, and soon became extremely laboured and anxious, attended by a loud and peculiarly harsh croaking sound, which was audible in every part of the hospital. A paroxysm of coughing occurred at intervals, and distressed the patient very much; it was abrupt, renewed rapidly for some seconds, and of a sharp thrilling kind, as of air forced with great impulse through a tense narrow passage. There was no expectoration, except that sometimes after great effort in coughing, a considerable quantity of colourless, ropy fluid would be suddenly thrown off, by an act of strong expiration, amounting nearly to vomiting. The patient's countenance was pale, and betrayed much distress. Pulse small, quick, and soft; temperature of the body rather low.

"The treatment of this severe paroxysm, consisted in the exhibition, at intervals, of the camphorated julep, with tinct. opii and æther; liberal demulcent drink, an epispastic to the thorax, and a light, warm cataplasm of flaxseed, chamomile, and spirit of camphor to the front of the neck, with bottles of hot water to the lower extremities. The symptoms of cynanche anginosa gradually passed off, and in 48 hours the patient was in her previous state, except some general debility. Paroxysms similar to the one just described were frequently renewed, commonly ensuing to some exposure or change of weather. In the course of three months she suffered not less than six or eight such attacks—usually of thirty-six or forty-eight hours' duration. —without any sensible impairment in the intervals of her general state of health; the paroxysms always breaking up with a discharge of large masses of uncoloured gelatinous matter. Great efforts were made to alter the state of parts which kept up the liability to those sudden attacks, with their train of terrible symptoms. The patient was put on a guarded mercurial course; small doses of calomel in combination with guaiacum and cicuta; with the employment, as liberally as possible, of tartarized ointment, over the upper region of the chest, and a succession of blisters kept open in front of the trachea; her diet was regulated, and bowels kept soluble. Once or twice her mouth was rendered slightly sore by the calomel, and during the continuance of that impression, the patient thought her breathing was much more free; but one or two severe seizures occurring after what was considered a full trial of mercury, it was withdrawn altogether, and various means afterwards employed. The plan of counter-irritation was frequently renewed, and having sometimes procured great advantage from the steady exhibition of copaiba, in the atonic forms of bronchitis and catarrh, an emulsion of that article was employed in the present case after the mercurial alterative experiment was given up.

"While under the balsam course, with counter-irritation externally, the patient had less cough, enjoyed longer immunity from acute attacks, breathed better, and was in many respects more comfortable; but still her voice was rough and stifled, and slight impressions of cold or damp brought on some degree of that harsh, stridulous, croaking kind of inspiration, which became so exalted and predominant in the severer paroxysms. On one occasion, when this woman had been in the yard of the alms-house, after a day of cold rain in August, she was affected at night with the usual symptoms of a suffocative attack. In this instance, the bad symptoms exceeded their common intensity; the usual palliatives, diligently applied, failed to give relief, and the patient expired suddenly, in twelve hours from the incursion of the paroxysm.

"*Examination, Eight Hours after Death.*—Along the anterior surface of the trachea, somewhat on its left side were a number, five or six, round bodies, from the size of a pea to that of a marble, lying loosely. Those bodies were of dark colour, part quite black; from their place and manner of distribution, they appeared to be the external bronchial glands degenerated. They were of soft pulpy consistence, the black bodies of the same colour within as on the outer surface. At the great division of the trachea, directly in the bronchial angle externally, appeared a round body, of dark red colour and considerable magnitude, about the size of an English walnut, the mass was dissected out, and felt firm and heavy. On attempting to cut through the middle of the body, its complete division was prevented by the resistance of a hard substance within, and a partial section only made. The body now presented a three-fold constitution; the external matter, for a quarter of an inch was dark red, then a layer of greater thickness, quite black, while the centre of the body was a hard, white, calcareous substance, supposed to weigh one drachm. The whole mass, like the bodies along the line of the trachea, seemed to be a morbid enlargement and conversion of the small glands usually occupying the external bronchial angle.

"The interior of the trachea was very pale, except just before its branching, where there was a florid patch, an inch in extent. The inner surface of the rest of the tube, up to the thyroid space, was not only without colour, but its mucous membrane so thin that it could not be certainly distinguished, and the cartilages seemed without any regular interior covering; nothing of the sort appearing, except a few filaments stretching across from one ring to another; yet there were no marks of distinct ulcerous waste in any part of the tube. There was no fluid of any kind in the trachea, until opening its primitive branches; these were found filled with the same ropy mucus which the patient had occasionally discharged in former attacks. The mucus found in the bronchial tubes was of brownish colour. The posterior part of the trachea, opposite the thyroid cartilages, was of almost cartilaginous firmness, and more than an eighth of an inch in thickness. The interior capacity of the trachea between the thyroid cartilages was very much contracted, leaving a triangular chink scarcely sufficient for the passage of a straw. The space of the larynx above the thyroid cartilages was also greatly narrowed, by thickening of the lining membrane, and more particularly of the reflection forming the rima, (the borders of which were hard and chord-like,) and the covering of the ventricular portion or appendages of the larynx.

"The lungs appeared sound. They were throughout expansible and crepitous, but heavy and dark-coloured, shewing great venous congestion. The patient died, probably rather of suffocation from the loaded state of the bronchial tubes and cells, complicated with strong spasmodic irritation of the larynx, than from any acute derangement or lesion. The mucus with which the bronchial passages were all filled, up to the great division, was so viscid that none of it would flow out by gravitation, and only small portions could be extracted mechanically."

The anatomical description of our author is not particularly clear, but still we think we gather enough to conclude that the organic lesion was not great. We regret that no mention is made of the state of the heart and large vessels, for if they were sound, we see no reason, on the face of the account of the dissection, why tracheotomy should not have been effectual in bringing relief to the breathing. The application of the stethoscope and employment of percussion is of much value in such cases, from the assistance they convey to us in ascertaining the condition of the lungs.

### II. AFFECTIONS OF THE HEART AND ARTERIES.

CASE 1. *Chronic Carditis—Hypertrophy with Condensation.* Ann Lee, æt. 26, tall and rather pale, admitted June, 1827.

"Temperature of the surface natural; skin soft; pulse 85, very small, not sensibly irregular or unequal; no fever or rigors; countenance pale; complexion a little sallow; eyes clear. Encephalon. No head-ache or vertigo; senses perfect; mind calm. Gastric system. Tongue clean; slightly lymphatic in the centre; no decided character of irritation; stomach not deranged in sensation; no morbid sensitiveness about the epigastrium; appetite commonly good; bowels regular; dejections scanty. Thorax. No cough; respiration small, but easy in general, the same when lying, sitting, or standing, but quicker after walking or any effort, and checked suddenly, but not painfully, on attempting full inspiration; no pain in the region of the heart, or the muscles of the chest or arm, and the patient could lie on either side, but preferred the left. Abdomen. Belly rather full, too prominent or rounded at the sides, slightly tense, not generally tender; sensibility greatest in the left hypochondrium, as if from a degree of splenitis, or of morbid tenderness in the left portion of the transverse colon; no defined hardness or volume in the part. This was the only acknowledged seat of soreness or pain about the body, and uneasiness, sometimes amounting to pain, was generally felt here, and frequently referred to by the patient. The region of the liver bore pressure well, which did not betray any unusual evolvement of that viscus. Pelvis. Menstruation suspended; no pain in that seat; urine scanty and high coloured, but voided without pain or irritation."

Dated the origin of her complaints to the preceding April, when her menses failed to appear, and since which time her health had declined. The patient was put upon an alterative course of calomel with squill, rhubarb, and canella—her mouth was slightly sore at the end of a week—the urine became free—and the œdema of the lower limbs nearly disappeared. The mercury was now discontinued, the bowels kept open with a bitter cathartic infusion, and after some time a pill composed of myrrh, assafœtida, turpentine, &c. exhibited with a view to excite the catamenial secretion. At the end of eight weeks she was so well as to walk about the wards and engage in her occupation of sewing, but after having been out of the hospital for some days she was attacked one evening by rigor, succeeded by fever, dyspnœa, acute pain in the upper part of the thorax, and cough. The pulse was very thick and thready, the patient much agitated, the orthopnœa distressing. Calomel and antimony with Dover's powder were exhibited, and the solution of tartarized antimony in barley-water. The patient passed a bad night, and on the next day was very weak with puffy face, quick thready pulse, inclination to stupor, cough less frequent, no expectoration. A large blister was ordered to the chest, calomel, camphor and ammonia every four hours, and wine whey drink prescribed. No improvement took place during the day, the orthopnœa increased to the utmost degree in the night, and after a few paroxysms of dyspnœa the poor creature sank down and expired suddenly.

" *Dissection.* The head was not examined. Thorax. Immediately under the upper third of the sternum appeared a considerable mass of recent gelatinous, or lympho-gelatinous deposit, of pale yellowish colour; the surface of the mediastinum and pleura, and for some space around, exhibited the appearances of recent inflammation; the serous membranes of the chest, (except the inflammatory patch described,) as well as the pulmonary surfaces, in their common state; the right lung much shorter than natural; the heart, with its envelope, presented very full in front, occupying the middle region of the thorax, rather than the left side, and appeared, (from the volume and seeming fullness of the pericardium,) to be larger than common; the front border or margin of each lung, was tied to the sides of the pericardium by three or four distinct, strong slips or bands, of fibro-ligament, of evidently ancient formation; the bottom of the left lung cohered extensively, by old adhesion, to the diaphragm, and in a partial degree to the pleura costalis; the parenchyma of both lungs was sound, soft, and crepitous; both pulmo-pleural sacs contained a few ounces of water; the heart, enclosed in its sac, being raised up, felt particularly firm and heavy, and retained its cordiform shape, as if its chambers were filled by some solid matter, while its exterior surface was closely embraced by the pericardium. On making a longitudinal section, to divide the pericardium and expose the heart, nothing of the common distinctness of parts could be found. The pericardium not only cohered to the heart, but was consolidated, or identified with its substance. This union was complete and universal up to the roots of the great vessels. The heart was weightier than natural, and fully equal to the medium size of the bullock's heart. The left ventricle was empty, and remarkably circumscribed, not capable, by conjecture, of containing more than half an ounce of fluid. Its thickness was rather more than an inch, its internal surface of natural appearance; the root of the aorta not sensibly hypertrophied; the semilunar valves soft and natural. The left auricle was of natural size, darker coloured than usual, firmer in its wall than common, but retaining a good deal its soft sacciform character; the mitral valve healthy. The right ventricle was smaller than common, less collapsed than in its natural empty state, no coagula in its cavity, its parietes very firm and thick, like the mass around the left ventricle; the root of the pulmonary artery and its valves sound. The right auricle was uncommonly prominent and rounded, and of particularly purplish-red colour, much darker than the rest of the heart. Its wall was half an inch thick in every part, its cavity did not collapse when cut open, and its whole interior was black and ragged, with short flocculent masses hanging from all the inner surface. The inside of the auricle represented a rough ulcerous cavern smeared and blackened with grumous blood. There were no traces of purulent or sanio-purulent fluid in the cavity, and only a few flakes of dark fibrinous matter. When the auricle was cleaned out, its interior surface still exhibited a perfectly ragged, (for want of a better term,) ulcer-like appearance. The tricuspid valve was found entire, but was undergoing calcareous degeneration. On being handled, its chordæ tendinæ cracked and broke to pieces between the fingers.

"Stomach healthy; remarkably small size, not more capacious than an equal extent of the colon. Spleen somewhat enlarged. The intestines and peritoneum natural, except a very blanched and bloodless appearance; some water in the peritoneal cavity, the abdominal aorta preternaturally small. The liver did not occupy more abdominal space than is common, but was twice its natural bulk; and from some cause not apparent, had ascended in the direction of the left thoracic cavity, pressing the diaphragm before it, and occupied fully half the space of that cavity; hence the compressed and shortened appearance of the left lung, before noticed. The liver, though hypertrophied, bore no particular mark of disease; the gall-bladder was small and contained a thin pale green fluid. The kidneys, uterus and bladder natural, the latter of very small size. The ovaries on both sides were converted into a mass of tuberculations, imperfectly suppurated. There were some remains of chronic ulceration in the vagina, the clitoris morbidly enlarged, and part of the labia interna demolished by former ulcerations. Both groins exhibited scars from ancient buboes."

Dr. Wright considers it remarkable that the carditis in the present case should have gone to such an extent without having caused fatal disturbance in the organ and general economy. Now to those who have seen much of these diseases there is nothing remarkable in the matter, on the contrary, it is an every-day occurrence. But we do not hesitate to say, and we speak it advisedly and from experience, that there *were* characteristic symptoms present, nay more, that the disease *ought* to have been discovered. The sallow puffy face, disposition to œdema, dyspnœa on exertion, pain on full inspiration, preference of one side on lying and tendency to orthopnœa, the small pulse and scanty urine are general symptoms that speak an intelligible language to those much accustomed to these cases. No mention is made of the chest having been stripped, or even of the hand having been applied to the region of the heart. We can say most conscientiously that we have not seen an instance of hypertrophy of the heart and adherent pericardium, in which the action of the organ was not seen and felt more extensively than natural. The stethoscope was not applied in the present instance, and yet we venture to predict that it would have indicated the existence of the hypertrophy with unerring certainty. We never examined such a case in which there was not powerful impulsion and the bruit de soufflet or the bruit de rape, and we have no doubt whatever that such would have been found in this instance, had they been looked for. No mention is made of the patient having suffered from rheumatism, but adhesions of the pericardium are very rare without it. Dr. Wright revives the exploded notion of Corvisart, that the *végétations* in the auricle were caused by the ancient syphilitic affection. We thought that the idea was long since abandoned by all sound pathologists. We might make many more remarks upon the case, but our limits compel us to forbear.

CASE 2. *Aortic Aneurism; Rupture into the Trachea and Œsophagus.*—Henry M'Claskey, æt. 54, very muscular, admitted December 1827.

"The leading symptoms, at the time of admission, were cough, and a constant sense of weight in the chest, increased on exercise, and causing labour of breathing after any considerable effort. The cough was hoarse and dry, without expectoration, not very frequent, not commonly excited or increased by deep breathing, the sense of weight in the chest constant, rather disagreeable than distressing, and not at all impeding lying down or walking about moderately. The patient represented his present symptoms to have come on about three weeks before, previous to which time he was, or believed himself to have been in good health, had been seldom sick, led an active life, and was free of any aptitude to cough, or other disorder.

"There was no fever, nor feverous temperature of the skin. Examined for many days together, the pulse betrayed no sensible fluctuation; it was sixty-five to seven; soft without volume, requiring pressure to distinguish it well, and not resisting with any energy of stroke; it was both a weak and sluggish pulse, though the latter is usually characterised by some force. The general state of the system corresponded with the torpor of the circulation; the man kept his bed, was silent, and seemed indifferent to every thing about him, his usual position supine, countenance dull and drowsy; when asked respecting his state of feeling, complained of annoyance by his cough, and of the sense of weight in his breast, spoke little, rather abruptly, and always in terms implying despondence of getting better."

The stethoscope was not applied, no diagnosis beyond "chronic pulmonary embarrassment" formed, and a palliative course of treatment, i. e. a negative one employed. Diarrhœa was prevalent in the infirmary, and the man became affected with it to the increase apparently of his cough. One morning he came out of the privy coughing very hard, violent vomiting was heard immediately afterwards, those present ran to the bed, where they found him ejecting blood in torrents, and in ten seconds' time he was lifeless.

"*Examination:*—No extravasated blood in the general cavity of the thorax. The right lung extremely dilated, filling the whole right cavity of the chest, of a deep purple hue, and engorged to the utmost possible degree, not from vascular congestion, but complete injection with blood, of all the bronchial passages and cells, to their minutest divisions; no artificial inflation of the lung could possibly have caused a more perfect display of its expansive capacity. The left lung was not at all dilated, and exhibited no unusual colour.

"The heart viewed in situ, gave but a very partial representation of the nature of lesion, some appearance of a pouch only presenting just beyond the arch of the aorta. The trachea and œsophagus were divided above and brought down, the membranous connexions around the thorax and to the spine separated, and the heart and lungs taken out together. Being now inverted, the state of the parts was readily traceable. At the deepest posterior part of the arch of the aorta, an inch and a half below the root of the left subclavian, was an aneurismal sac, the size of an egg, its parietes soft and apparently very thin. This was plainly the source of the hæmorrhage, and to trace its communications, the sac was slit open through its greatest length. The coats of the artery, (within the limits of the sac,) were very thin and tender, and tore rather than cut when laid open; the sac was empty, except a few delicate layers of soft coagulable lymph. Passing the finger into the sac, it encountered three or four hard, rough, pointed bodies, on each side, within the aneurismal cavity, which were the extremities of three broken rings of the trachea; the points were thin and sharp, as if wasted, and had a roughness, hardness, and brittleness, more of bone than cartilage. The communication with the trachea, and the cause of bloody insufflation of the right lung, were thus explained; it remained to ascertain why the fatal hæmorrhage had occurred in the form of a violent and repeated gush by vomiting. When the œsophagus was now detached from the trachea behind down to the borders of the aneurismal sac, it was found to be united by adhesions both with the diseased portion of the trachea and a part of the aneurismal bag; a farther separation of the œsophagus from the trachea, disclosed an oval opening, in the former, large enough to admit the point of a finger, by which the œsophagus communicated with the trachea, just behind where the rings of the latter had given way, which was pretty low on the left side of the trachea. The coats of the œsophagus were very much attenuated over the whole extent of its adhesion to the trachea and sac, and the rent described communicating directly with the current flowing into the trachea after rupture of the rings; the blood seems to have passed freely also, by the route to the stomach; hence its discharge by distinct acts of full vomiting. The stomach contained after death about a pound of coagulated blood.

"It has been mentioned that the left lung was not dilated, or changed in colour, &c. The cause of this difference in the two lungs, was explained while removing the parts from the cavity of the chest. The left lung was found to adhere with great firmness throughout its whole anterior, lateral, and posterior surface, to its own pleura, and by that to the serous membrane of the ribs. The lung was enlarged very much, firm and heavy, and in its whole substance hepatized to so great a degree, that every trace of bronchial tubes and cells was wholly obliterated up to the point where the left bronchus penetrates the lung by its primary branches. From this point there was no channel by which air could enter the lung, and for that cause, the extravasated blood was totally excluded."

This interesting case adds another to the many already upon record, that stamp the insidious character of aneurismal dilatations of the aorta. As Dr. Wright very properly observes, no dependence can be placed upon the statements of previous good health which patients very generally give, for it is obvious in the present instance that the hepatization of the whole of the left lung must have been an alteration of considerable date. It may be asked if the stethoscope would have pointed out the real nature of the disease. It would have shewn the hepatization of the lung beyond a doubt, and we think that in all probability it would have indicated something wrong about the aorta. In dilatations of the arch with any degree of hypertrophy of the left ventricle, we almost invariably have a louder sound, or even a decided bruit, above the right clavicle, and in all the cases of aneurism of the aorta that we have examined there has been this indication in the situation of the tumour. On this point, however, we would be understood to speak with some diffidence as we have not examined a sufficient number of cases of aortic aneurism to justify us in forming a sweeping conclusion. With the succeeding case we must conclude.

Case 3. *Gangrene of the left upper extremity—peculiar affection of the arteries.*—Margaret Cash, æt. 56, admitted Oct. 20th, 1827, with aggravated diarrhœa, but no symptoms betraying urgent danger. The left hand was more powerless than the right, and the pulse in the former was barely perceptible. The diarrhœa was much moderated by judicious remedies, but a new train of phenomena now presented themselves.

"Ever since the patient's admission into the infirmary, she had complained of pain in the inner side of the left arm, a little below the insertion of the tendon of the teres major. Nothing appeared at the place indicated, to explain the cause of pain there; the part was sore to the touch, but neither swelled nor inflamed; the soreness extended an inch or two up and down the arm, in the track of the brachial artery. At the end of a week from her entering the ward, the pain of the arm, and soreness to handling, had almost entirely disappeared, but the patient was then sensible of total loss both of power of movement and sensation in the left hand. That hand was quite cold, and on the tenth day a faint bluish tinge was discovered over all the fingers, the colour permanent, and not varying by pressure. The discoloration of the fingers became deeper every day, spread slowly, first up the back of the hand, then through the palm, and by the sixth day from its appearance, had reached the carpal articulation all round. The fingers, up to the metacarpal junction, were now evidently lifeless; they were cold, black, and unpliant, yet not at all sphacelated, nor showing any vesiculation, or other marks of putrescent decay; nothing like separation of parts was manifested ; the blackness was lost indefinitely, by a fainter shade in the adjacent skin. The forearm next showed the same character of lividness, first occurring to the fingers ; but on the arm the expression was different ; here it appeared in the form of purpura, in blotches at distinct points on the arm, chiefly on the back of the arm, from the wrist to the small head of the radius. The blotches were large, irregular in form, and in a few hours after their appearance some of them became slightly vesicular or bullous, the cuticle being somewhat raised by sero-sanious extravasation. During this change in the condition of the arm, there were no marks of that low, topical, inflammatory action, which accompanies gangrena sphacelus; the arm neither swelled nor became hot. The death of the limb seemed to be taking place in a manner purely passive.

"As soon as the livid tinge was perceived on the fingers of the left hand, the patient, notwithstanding the continuance of general feverous irritation, was put on a decidedly sustaining course ; the quinine was exhibited freely, with the cordial mixture, (sub. carb. ammoniæ, aq. menth. et tinct. cardamom. so much commended by Mr. Cooper ; ) opium and camphor were given at night, the first liberally ; the hand and arm were kept enveloped in fomentations of bark and chamomile, charged with spirit of camphor and tincture of myrrh. The supporting plan, prosecuted regularly for many days, and varied according to circumstances, made no satisfactory impression. The powers of life gradually declined, stupor supervened, and the patient expired on the twenty-first day after entering the hospital. The discolouration of the left arm never spread above the elbow-joint, and nothing of gangrene, nor any petechial or purpurous spots appeared on any other part of the body ; there was no gangrenous fetor about the arm. For more than a week before the death of the patient, the diarrhœa was inconsiderable.

"*Examination.*—For the purpose of tracing better the state of the arteries in the left arm, a pipe was introduced into the left subclavian, and the fine injection thrown in as fully as possible ; the arm was then removed at the shoulder-joint. The artery and vein, (cephalica magna,) had a very unusual relation to each other. The former was filled with the injection as far as the bend of the arm ; its course was very serpentine, and two inches below the root of the superior profunda, made a sudden curve, so as to describe a semicircle an inch in diameter. This bend was apparently referrible to the state of the vein at that point. From the axilla downwards, the great vein was enlarged, and filled with semi-solid black blood ; in two or three places above the doubling in the artery, the vein was dilated into distinct circumscribed tumours of moderate size, while at the place of sudden curvature in the artery, there was one of those tumours the size of a musket ball, and solid as if the contained blood was firmly coagulated. Round this great varix the artery turned close, to take afterwards its ordinary course to the bend of the arm. It was to this spot the patient pointed as the seat of pain in the arm. The external cellular coat of the artery exhibited a high red colour throughout its whole course, from the axilla to the bend of the arm ; just above the turn of the artery round the great varix, its thickness was increased for the space of an inch, to more than three times its circumference in any other part. This enlarged portion of the artery was firm, and consisted not in aneurismal dilatation, but in general thickening of its coats all round, to one-third of an inch, by which its channel was very much diminished. The profunda superior was very much enlarged in its trunk, and involved at its branching in a plexus of veins, showing a number of varicose pouches filled with black blood.

"The roots of the radial, ulnar, and interosseal arteries, were full, round, and hard, apparently distended by injection ; but it was found that the injection had not penetrated below the bend of the arm, and was stopped there abruptly by a plug of solid coagulum, which extended continuously through every trunk and considerable branch of the forearm.* There was no earthy deposit in the coats of the humeral or proper brachial arteries.

"The right arm was dissected without previous injection; the veins were large, but natural in appearance; no varices or other irregularity. The humeral artery, with an exception to be noticed directly, was of ordinary appearance, and regular in its course. At two fingers breadth below the root of the profunda, the artery was suddenly enlarged into a bulbous body, somewhat oval in shape, and about the size of a large almond; its walls very thick and spongy, with portions of loose flaky matter in their substance; the centre of the tumour contained a little pus, and immediately around the pus was a substance resembling the matter of crude tubercles. The enlargement of the artery then was the effect of steatomatous degeneration in its coats, and the tumour which had been found in the left humeral artery was the same morbid conversion, less advanced. The canal of the right artery was not quite obliterated at the point of disease; it was pervious there, but the passage very contracted, and not through the centre of the tumour, but on the side next the bone, and very near the surface. Although the continuity of the tube was preserved, it is probable that little if any blood passed through the diseased part of the artery; the passage through the tumour was very confined, and the trunk of the artery, directly above and below the enlargement, had a shrunk and wasted appearance. The profunda was very full in its trunk, and divided near its root into five principal branches, three of the largest taking a course down the arm. It is probable that the forearm depended mainly, if not altogether, on the supply by the branches of the profunda, through the anastomotics; the state of the latter branches could not be satisfactorily ascertained, as the arm had not been injected. The cellular coat of the right humeral artery had nothing of that strong red tinge, which was very obvious over the whole trunk of the artery in the left arm.

"The heart was much enlarged and flaccid; the right auricle uncommonly capacious; the descending and ascending cavas dilated in a very remarkable degree, before entering the auricle. The right ventricle was dilated, and its wall thin; the root of the pulmonic artery expanded in a pouch-like form, its coats attenuated, and the central points of its semilunar valves chalky. The left auricle as usual. The left ventricle large, flaccid, and thin. The coronary arteries hard and friable. The root of the aorta much dilated; at the arch swelled into a bag-like expansion, twice as capacious as the healthy form of the vessel at that point; its semilunar valves full of concretions. The enlargement of the aorta, more regular in figure, continued down the trunk through the chest and abdomen, and the whole track of the vessel, from the ventricle to the bifurcation, was studded with calcareous formations in large patches, smooth and firm, and as usual, lying between the coats. At the arch, the patches of calcareous matter were particularly large, two or three of the plates equal in surface to a twenty-five cent piece, and exceeding an inch in length. The carotids and subclavians did not show any degeneration of their coats; the veins of the neck were uncommonly large and distended with black blood. The lungs were sound, but unusually dark and heavy, from engorgement of the pulmonary veins. In the abdominal cavity there was no manifest lesion of any of the structures; the mucous coat of the small intestines showed some patches of slight phlogosis. The encephalon was not examined.

"The appearance of the inferior extremities was natural."

We think with Dr. Wright, that the

---

* "The humeral artery was dissected out of the arm, and with it an inch or two of the branches in the forearm containing the coagula described; the concreted blood could not be pressed out, and now after twelve months maceration in dilute alcohol, the coagulum is perfect as ever, showing a deep purple hue of the branches as far as it extends."

gangrene of the left upper extremity depended on constantly failing energy in the whole limb rather than on any particular obstruction. A woman of fifty-six, with a flaccid heart, extremely morbid arterial system, and worn down by diarrhœa, is no unlikely subject for gangrene of an extremity, especially if any local peculiarities of the blood-vessels should concur in diminishing the vital energy of the part. The mortification in the present instance had much of the character of that "dry gangrene" which occurs after sudden obstructions of the circulation in a limb, or in very enfeebled constitutions. The case is interesting in a physiological point of view, and also in reference to what we may term the morbid anatomy of advanced age, that period so exposed to danger from the wear and tear that the passions and the turmoil of life have inflicted on the heart and arteries.

